# Cognitive Impairment Associated With Schizophrenia: New Research Agenda

**DOI:** 10.1093/schizbullopen/sgae023

**Published:** 2024-09-19

**Authors:** Silvana Galderisi, Stephen R Marder

**Affiliations:** Department of Mental and Physical Health and Preventive Medicine, University of Campania Luigi Vanvitelli, Naples, Italy; Department of Psychiatry, Semel Institute for Neuroscience at UCLA and the VA VISN 22 Mental Illness Research, Education, and Clinical Center, Los Angeles, CA, USA

The Special issue of *Schizophrenia Bulletin Open* focusing on Cognitive Impairment Associated with schizophrenia (CIAS) highlights recent progress in a clinical and research field that remains rarely assessed and addressed in clinical practice, despite many statements on its key role in the pathogenesis, course, and outcome of schizophrenia.

The main factors explaining this paradox include (a) the irrelevance of CIAS for the diagnosis of schizophrenia (it is not part of the diagnostic criteria in either DSM 5 or ICD 11); (b) the belief that it is just an epiphenomenon of psychosis that can be addressed by treating the psychosis, (c) the assumption that nothing can be done to treat it, and therefore there is no reason for assessing it, and (d) clinicians often lack the time and few have access to trained personnel for the assessment of cognition.

Most of these beliefs are in contrast with the available evidence. In fact, it is largely acknowledged that CIAS is independent of other psychopathological domains^1^ and has trait-like characteristics: it is observed in all stages of schizophrenia, in subjects at clinically high risk for psychosis, and in non-affected first-degree relatives of persons with schizophrenia. It has been suggested as an intermediate phenotype candidate indexing liability to schizophrenia.^2^ There is evidence that deficits in multiple neurocognitive and social cognition domains interfere with real-life functioning more than negative and positive symptoms.^1^ Furthermore, psychosocial interventions, such as cognitive remediation and physical exercise, provide consistent benefits, especially when integrated into a structured psychiatric rehabilitation program, as attested by several meta-analytic studies.^3^ Although currently available pharmacological treatments for schizophrenia have little or no efficacy for CIAS, their correct use (eg, avoiding the use of high doses of first-generation antipsychotics or anticholinergic drugs) may prevent further deterioration. In addition, phase III clinical trials of new drugs suggest that soon we might have medications with an indication for CIAS.

The lack of trained personnel for assessing cognition is an important concern. It is well known that in many countries, mental health services are understaffed, and often the available staff does not include personnel trained in the assessment of cognition in patients with schizophrenia. As a result, the solution to this problem may require training front-line providers including psychiatrists and psychologists to assess patients. Tools for the assessment of CIAS, even the current gold standards, are, in fact, mostly borrowed from other branches, and therefore might not represent the ideal tools for both people with schizophrenia and mental health care providers.

Moving from consolidated evidence of the key role of CIAS for schizophrenia treatment and functional outcome, the Special Issue of *Schizophrenia Bulletin Open* on “*Cognitive impairment associated with schizophrenia: new research agenda*” aims to provide an update on recent developments in the field, examine aspects previously unaddressed, and highlight gaps requiring further research.

As highlighted above, apart from the need for educational programs aimed to contrast the erroneous beliefs about CIAS, the shortage of time and trained personnel plays an important role in the attitude to ignore CIAS in most clinical settings. The uptake in clinical practice of different assessment tools, developed ad hoc, might overcome this barrier, provided that their validity is proven across different settings and cultures. The papers by Pezzella et al^4^ and by Horan et al^5^ highlight alternative methods for the assessment of CIAS. The former moves from the consideration that standardized neuropsychological assessment represents the gold standard in research on cognitive impairment but has never become part of the assessment in clinical settings. Interview-based cognitive assessments might be less time-consuming and more practical and easier to use in those settings; they might provide patients and caregivers with better insight into the presence of cognitive impairment and its impact on real-life functioning and may increase motivation to participate in cognitive rehabilitation programs. Based on these considerations, the study aims to provide further validation of the Cognitive Assessment Interview (CAI)^6^ for assessing cognition in a large sample of community-dwelling patients with schizophrenia. The paper demonstrates that the assessment of cognition using the CAI or the MATRICS Consensus Cognitive Battery (MCCB) leads to similar results on the impact of cognitive impairment on real-life functioning and on possible mediators of this impact. Horan et al, openly question the use of traditional neuropsychological tests to assess CIAS and propose “ecologically focused measures,” that is, measures designed to more closely capture the essence of everyday cognitive skills as they are used in real-life situations. Measures of functional capacity do represent ecological tasks, as they assess the ability to perform everyday living skills in controlled situations and, in fact, show a stronger and more direct link to real-life everyday life skills than neurocognitive and social cognition domains assessed by traditional neuropsychological tests. However, as noted by Horan et al, these measures share several challenges and limitations with traditional cognitive tests, including training demands, artificial experimental context, and outdated if not obsolete content. In their paper, Horan et al provide a comprehensive review of two promising newly developed approaches: one based on the use of technology to enhance the ecological validity of cognitive and functional assessments, including technology-enhanced performance-based measures of functional cognitive skills, and the other one based on remote cognitive and functional assessments using Ecological Momentary Assessment (EMA) and passive metrics relevant to schizophrenia.

Berg et al^7^ addresses the relationship between cognition and negative symptoms. Using a transdiagnostic approach that includes both bipolar I and schizophrenia subjects, they explore the relationship between domains of negative symptoms and working memory. They find that avolition-apathy—among the most important domains of negative symptoms—is related to impairment in working memory. Moreover, this relationship is present in both the bipolar 1 and the schizophrenia patients. The results underline the importance of evaluating cognition in patients with motivational disturbances.

Another paper in this special issue^8^ focuses on the complex relationship between cannabis use and the emergence of schizophrenia. Although cannabis users with schizophrenia tend to have worse symptoms and functional outcomes than non-users, there is a paradoxical finding from a number of studies indicating that users perform better on cognitive tests. Lesh et al use task-based functional Magnetic Resonance Imaging (fMRI) to explore dorsolateral prefrontal cortical (DLPFC) recruitment in cannabis and non-cannabis users with schizophrenia and controls. They find both higher DLPFC recruitment in the users as well as higher levels of cognitive control. This and other studies raise the intriguing possibility that cannabis users include individuals who may not have developed psychotic illnesses if they had not used cannabis.

The paper by Vita et al,^9^ provides a critical review of current and future perspectives on pharmacological treatments for CIAS. Moving from the consolidated evidence that antipsychotic drugs have little or no impact on CIAS, and may even contribute to its deterioration, the paper highlights the importance of accurate management of antipsychotic medications, including avoiding additional treatments that can further exacerbate CIAS. The paper provides a very informative update on molecules that are currently being investigated in phase 2 and phase 3 trials, have already provided very promising results, and might represent the awaited pharmacological game-changer in the treatment of CIAS.

The review by Joshi^10^ focuses on a different approach to treatment, that is, Targeted Cognitive Training (TCT). This is a “bottom up” approach to cognitive training that focuses on improving lower-level processes, such as auditory or visual processing, that may underlie the deficits in higher-level cognitive processes. The review also discusses the opportunities for a precision medicine approach that would use biomarkers to identify individuals likely to respond to a particular intervention. For example, measures of auditory processing speed collected during the first hours of TCT may be useful for identifying responders.

This special issue of *Schizophrenia Bulletin Open* underlines the importance of understanding and evaluating cognition in people with schizophrenia. New findings can improve our understanding of the relationship between CIAS and the outcome of schizophrenia. In addition, new approaches to pharmacological and non-pharmacological treatment indicate that there are new opportunities for addressing these impairments.
